# Energetic consequences of prey type in little penguins (*Eudyptula minor*)

**DOI:** 10.1098/rsos.221595

**Published:** 2023-08-30

**Authors:** Natalie Petrovski, Grace J. Sutton, John P. Y. Arnould

**Affiliations:** ^1^ School of Life and Environmental Sciences, Deakin University, 221 Burwood Hwy, Burwood, Victoria 3125, Australia; ^2^ Department of Environment and Genetics, and Research Centre for Future Landscapes, La Trobe University, Bundoora, Victoria 3086, Australia

**Keywords:** foraging behaviour, seabird, optimal foraging, handling time

## Abstract

Investigation of foraging decisions can help understand how animals efficiently gather and exploit food. Prey chase and handling times are important aspects of foraging efficiency, influencing the net energy gain derived from a prey item. However, these metrics are often overlooked in studies of foraging behaviour due to the difficulty in observing them. The present study used animal-borne cameras to investigate the type, duration and energetic consequences of predator–prey interactions in little penguins (*Eudyptula minor*) (*n* = 32) from two colonies in Bass Strait, south-eastern Australia. A total of seven main prey items were observed and consumed by little penguins. Penguins were observed to consume prey types and use strategies that have not been previously documented. These included consumption of bellowsfish (*Macroramphosus scolopax*) and other fish species captured sheltering around jellyfish or extracted dead from the tentacles. Chase and handling time varied with prey type and lasted approximately 2 s for most prey. Profitability varied among prey types, with a greater amount of low profitable prey being consumed, suggesting a trade-off between minimizing energetic costs, and increasing capture rates. These results highlight the use of animal-borne video data loggers to further understand the foraging adaptations of important predators in the marine environment.

## 1. Introduction

An important factor influencing optimal foraging is the time that it takes to locate, chase, capture and process a prey item as this can impact its profitability [1]. Handling time has been defined as the duration of contact with a prey item, including capturing, manipulating and ingesting [2]. Prey chase and handling times can play a crucial role in prey selection and, therefore, can influence search efforts and impact energetic intake. Handling time is not always constant and varies with prey-specific characteristics such as the size, type and prey abundance [3–5]. Furthermore, handling time may vary due to predator related attributes such as the level of satiation of the predator or the presence of any other predators [6–8].

Fine-scale foraging behaviours are generally more difficult to observe in free-ranging cryptic species as a lot of their foraging occurs either discretely, rapidly or beyond observer visibility [9,10]. In particular, there has been difficulty in investigating underwater feeding behaviour in many marine species [11]. Early prey consumption studies include use of accelerometers, regurgitations and stomach flushing [12–14]. However, these methods make it difficult to explain how animals allocate their time foraging at sea. It is only recently, with advances in technology, that fine-scale foraging behaviour has been able to be recorded in marine top predators (e.g. [15–17]). Early camera equipment was complex, expensive and large, but the improvements in biologging technology and miniaturization of devices in the last two decades have allowed us to directly visualize these foraging behaviours [18].

Seabird species have adopted different foraging strategies based on morphological adaptations and marine physical features [19]. Marine ecosystems are vulnerable to rapid changes in diversity and function and many seabirds are influenced by changes to their environment [20]. Bass Strait, in south-eastern Australia, is an important area for seabirds, supporting approximately 20.5 million individuals of at least 18 different species [21]. The region has experienced some of the most rapid increases in oceanic temperatures worldwide, with the anticipated changes expected to alter the abundance, diversity and distribution of marine prey [22,23]. Therefore, understanding the factors influencing foraging behaviour and success in marine predators in this region is crucial for the effective management and long-term conservation of their ecosystems.

Little penguins (*Eudyptula minor*) are ubiquitous seabirds in south-eastern Australia and are significant consumers of marine resources [24]. The highest concentration of little penguins is in Bass Strait with an estimated 85 000–151 800 breeding pairs [25] with the annual food requirements of little penguins in Bass Strait estimated to be 37 000^3^ kg [14]. They are considered to be generalist and opportunistic feeders, with diet variation reflecting food resource availability and breeding stages [26,27]. The oceanographic variability in Bass Strait is known to influence little penguin foraging and breeding success [28]. Consequently, a reduction in prey, by natural means or human activities, would have a serious effect on survival and reproductive success [29]. While foraging behaviour in little penguins has been extensively documented (e.g. [30–34]), some aspects of their foraging strategies are not well understood [20]. In particular, little is known of the factors which influence the time spent foraging and interacting with prey items and the possible reasons for their foraging decisions [35]. Furthermore, there is little information on prey chase and handling times and the factors influencing these behaviours. Such knowledge is important to understand how the species may respond to environmental change [20,36].

The aims of the present study, therefore, were to investigate in little penguins: (i) the factors influencing prey chase and handling times and (ii) the energetic consequences associated with these durations.

## 2. Material and methods

### 2.1. Study sites and animal handling

To maximize the variability of prey types encountered, fieldwork was conducted at two locations that differ in levels of marine primary productivity: London Bridge (LB) (38.62° S, 142.93° E) and Gabo Island (GI) (37.56° S, 149.91° E) in south-eastern Australia. The LB little penguin colony is located on the mainland coast in western Bass Strait at the base of sandstone cliffs and is comprised 100–150 individuals. GI is located approximately 0.5 km from the eastern coast of Victoria and, at the time the data were collected, supported the second largest known colony of little penguins in Victoria with approximately 15 000–18 000 breeding pairs [37].

The study was conducted during the chick-rearing period in 2015 and 2016 (October–November). Nests with chicks in guard stage, when partners alternate foraging days and one parent is always present, were selected [38]. Adults were captured by hand from their nest burrows before sunrise. Birds were weighed in a cloth bag using a spring balance (±10 g, Super Samson, Salter Brecknall, UK) and morphometric measurements of bill depth (BD), bill width (BW), bill length (BL) and head length (HL) were taken using Vernier callipers (±0.1 mm). Flipper length (FL) was recorded using a ruler (±0.1 mm). Sex was determined from BD measurements following the methods of Arnould *et al*. [39].

Individuals were equipped with a miniature video data logger (BirdCam, Catnip Technologies, 25 × 45 × 15 mm, 24 g, 400 × 400 pixels at 28 frames per second) programmed to record on a duty cycle of 30 min on : 30 min off. As part of concurrent studies, individuals were also instrumented with a Global Positioning System (GPS) data logger (IgotU120, Mobile Action Technology, 44.5 × 28.5 × 13 mm, 17 g including waterproofing and tape) and an accelerometer/dive behaviour data logger (AXY-Depth, TechnoSmArt, 12 × 31 × 11 mm, 6.5 g). Data from these additional devices are not reported here. The video data logger and GPS were sealed in heat shrink tubing for waterproofing. All devices were attached to the dorsal feathers with waterproof tape (Tesa 4651, Germany), positioned along the midline of the lower back, with the camera facing forward and in front of the other units to streamline the package to minimize hydrodynamic drag. Together, the devices weighed less than 48 g, approximately 4.2% of the average body mass of little penguins in this study (1148 ± 15 g). A uniquely numbered passive integrated transponder tag (PIT tag, Trovan, 11 × 1.5 mm) was then implanted subcutaneously between the scapula before individuals were returned to the nest burrow to resume normal behaviours and from where they voluntarily left for a foraging trip. Individuals were then recaptured after a single trip to sea and the devices were removed by peeling off the waterproof tape from the feathers. Efforts were made to minimize handling time with all procedures lasing less than 10 min whilst the individual remained in a cloth bag for the entire duration.

### 2.2. Data processing and analysis

The video from the data loggers were analysed frame-by-frame using the behavioural coding program *Solomon Coder* [40]. Where evident, each frame categorized: water column behaviour (included diving, surface, preening and transiting); prey item present; prey abundance; foraging behaviour (included chase, strike and handling time); conspecific and heterospecific present and conspecific and heterospecific abundance. Fish were identified using fish identification websites, Fishes of Australia and FishBase [41,42]. Fish were classified to the lowest taxonomic level possible. A chase was defined as intentioned movements, often with an increase in swimming speed towards a prey item. A strike occurred when the individual attempted to capture the prey and handling time was recorded as time from capture to complete consumption or discarding of the prey. The sum of the chase, strike and handling times are hereafter referred to as prey encounter time. Individuals where prey capture events were not observed during video recording periods were excluded from further analyses.

To investigate the factors influencing prey encounter time, Linear Mixed-Effects Models (*lme4 package*, [43]) based on the restricted maximum-likelihood method were constructed in the R statistical environment [44]. Prey encounter time was modelled against predictor variables of prey type, sex and morphometric measurements (body mass, FL, BL, BW and HL). Bird identity was included as a random effect to account for repeated measures and to obtain an estimate of individual variation. Collinearity was assessed by inspecting correlation coefficients (Pearson correlation, *r* ≥ 0.7 or ≤−0.7), with all predictor variables remaining in the modelling process. Model comparison and averaging, using the ‘dredge’ function within the *MuMIn* package [45] were undertaken to rank candidate models based on Akaike Information Criterion scores (AICc). Prey encounter time was log-transformed as this data was not normally distributed. Candidate models were identified as those with a ΔAIC of less than or equal to 4 and used to generate model-averaged coefficient estimates. Between-subject variance (*τ*_00_) was calculated using the package *insight* [46] to assess how much the individuals differed from each other in order to inform if the random factor was affecting the prey encounter model results. Tukey's *post hoc* testing was performed on models using the *agricolae* package [47] to determine differences between encounter durations of prey types.

Estimates of gross energy content of prey (GE; kJ) were obtained from published values of mean mass (g) and energy content composition (kJ·g^−1^) of identified prey types (table 1). To estimate the energy content of unidentifiable prey, the mean of all commonly consumed prey types was calculated. Energy expenditure during prey encounters was estimated by identifying the power requirements of little penguin underwater swimming at sea at their preferred speed, which was predicted to be a value of 0.02 (kJ kg^−1^ s^−1^; [54]. Foraging efficiency (FE, kJ·s^−1^) for each prey type was then calculated as

$$\mathrm{FE}=\;\frac{\mathrm{GE}-\mathrm{EE}}{\mathrm{FT}},$$

where GE is the gross energy content of the prey item, EE is the energy expended during the prey encounter (chase and handling) and FT is the duration of the encounter.

**Table 1.** Estimated energy content of prey consumed by little penguins (*Eudyptula minor*) in video recordings.

prey types	mean mass (g)	energy content (kJ·g^−1^ wm)	GE (kJ)	references
bellowsfish (*Macroramphosus scolopax*)	2.41	3.9	9.4	Froese & Pauly [42]; Martins *et al.* [48]
juvenile *Clupeiformes*	0.92	2.2	2	Froese & Pauly [42]
prey captured from jellyfish	3.17	3.92	12.4	calculated mean of prey types observed swimming in or around jellyfish—*Macroramphosus scolopax*, juvenile *clupeiformes* and *Trachurus spp*.
Australian anchovy (*Engraulis australis*)	5.5	5.2	28.6	Cullen *et al.* [49]; Bunce [50]
jellyfish (*Cyanea spp*)	2.88	0.4	1.2	Doyle *et al.* [51]
mackerel (*Trachurus spp*)	6.19	5.65	35.0	Froese & Pauly [42]; McCluskey *et al.* [52]
pilchard *(Sardinops sagax)*	6.5	8.6	55.9	Cullen *et al.* [49]; McCluskey *et al.* [52]
unidentified baitfish	4.70	5.1	24.0	calculated mean of small schooling fish already identified and commonly observed with similar handling times—*Macroramphosus scolopax, Trachurus spp., Engraulis australis*
other prey	4.50	6.1	27.5	calculated mean of identified prey types with minimal occurrence, grouped into one category/Cullen *et al.* [49]; Gales [53]
unidentifiable	3.94	4.27	16.8	calculated mean of all identified prey types

## 3. Results

### 3.1. Prey events

Foraging trip video data were obtained from a total of 32 individuals, 23 from GI and 9 from LB. A further 25 deployments were excluded from analyses (15 from GI and 10 from LB) due to either the individual not heading out to sea, no foraging activity observed (e.g. device capturing departure from colony only) or malfunction of the devices. All tagged birds returned to their nests after a single foraging trip and all devices were retrieved. The average mass change from deployment to retrieval was a gain of 4 ± 1.2 g, however, individuals were not strictly captured before feeding their chicks. A combined total of 102.9 h of video data were obtained with a mean duration of 3.12 ± 0.2 h per individual (range 0.25–4.5 h). Due to the duty cycle, the recorded data sampled over approximately 7–8 h, representing an estimated 50% of the foraging trip duration [31]. During these video recordings, a total of 2078 prey events were identified, with the majority observed at GI (96.4%). Sample size and video data duration were considerably less at LB, contributing 3.6% of total prey events.

**Figure 1.**
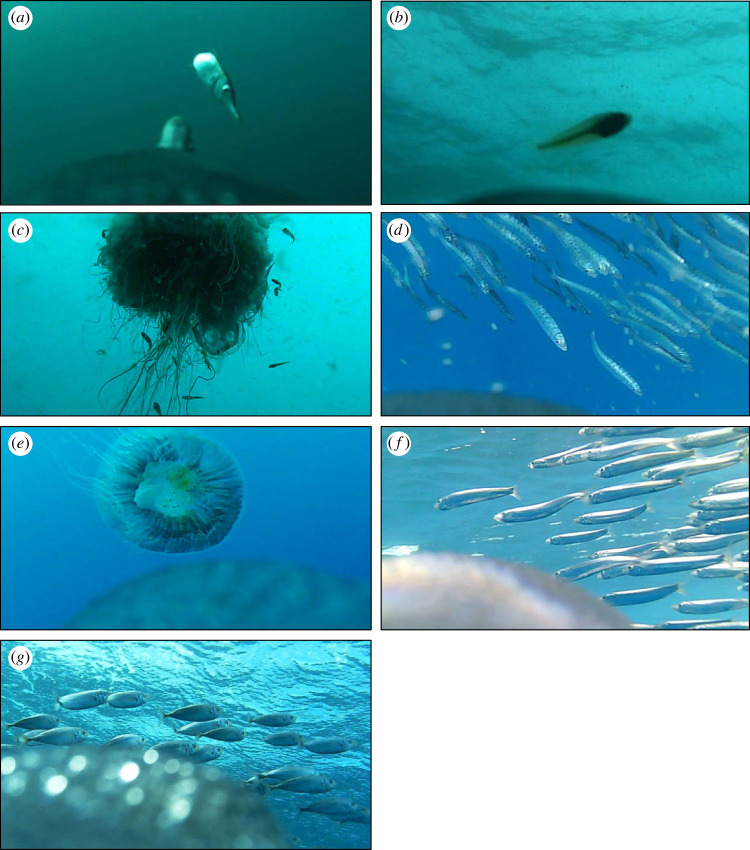
Representative images from animal-borne cameras of prey captured by little penguins, including: bellowsfish *Macroramphosus scolopax* (*a*); juvenile clupeiformes (*b*); prey captured from jellyfish (*c*); pilchard *Sardinops sagax* (*d*); jellyfish *Cyanea spp.* (*e*); anchovy *Engraulis australis* (*f*); mackerel *Trachurus spp.* (*g*).

Seven main prey types were captured by little penguins: bellowsfish (*Macroramphosus scolopax*); juvenile *Clupeiformes*; Australian anchovy (*Engraulis australis*); jellyfish (*Cyanea* spp.); mackerel (*Trachurus* spp.); pilchard (*Sardinops sagax*) and prey captured from jellyfish (fish species sheltering in or around jellyfish including bellowsfish, clupeiforms and mackerel; figure 1). Additional prey types were observed but were grouped into a single category (other prey) due to their infrequency, with less than 10 captures in total. Species identified in this group included: Gould's squid (*Nototodarus gouldi*); blue warehou (*Seriolella brama*); sandy sprat (*Hyperlophus vittatus*); garfish (*Hyporhamphus melanochir*); pipefish (*Syngnathinae* spp.) and whiting (*Sillaginidae* spp.).

Due to many captures occurring out of camera view or not being clearly visible, several prey items could not be identified and were grouped as either unidentifiable (20.0%) or unidentified baitfish (small schooling fish that have already been observed and fit similar descriptions to bellowsfish, anchovy and mackerel, 12.3%). All observed prey types were encountered by little penguins at GI, of which bellowsfish were the most consumed prey accounting for almost a quarter of all captures (24.4%, table 2). By contrast, at LB, bellowsfish and mackerel were not encountered, with the most encountered prey here being anchovy and pilchard (18.7% and 16% at LB, respectively). Prey captured from jellyfish was the second most consumed prey type (14.9%), with almost all occurrences at GI. Jellyfish also contributed to a high proportion of prey captures (9.2%), again with most captures occurring at GI. Juvenile clupeiforms were one of the least consumed prey types among both sites (0.7%); however, this does not include the identified juvenile clupeiforms observed in the captured from jellyfish grouping.

**Table 2.** Proportion of prey captures by little penguins at London Bridge and Gabo Island and their mean encounter times.

prey type	captures (*n*)		proportion of captures (%)	median prey encounter time (s)
	GI	LB		
Australian anchovy (*Engraulis australis*)	212	14	10.9	1.07
bellowsfish (*Macroramphosus scolopax*)	506	0	24.4	1.07
juvenile *Clupeiformes*	14	0	0.7	0.82
mackerel (*Trachurus spp*)	125	0	6.0	1.18
jellyfish (*Cyanea spp*)	189	2	9.2	3.18
pilchard (*Sardinops sagax*)	4	12	0.8	1.52
prey captured from jellyfish	309	1	14.9	1.32
unidentified baitfish	256	0	12.3	1.07
other prey	17	2	0.9	1.25
unidentifiable	371	44	20.0	1.25
total	2003	75		

### 3.2. Prey encounter time

**Figure 2.**
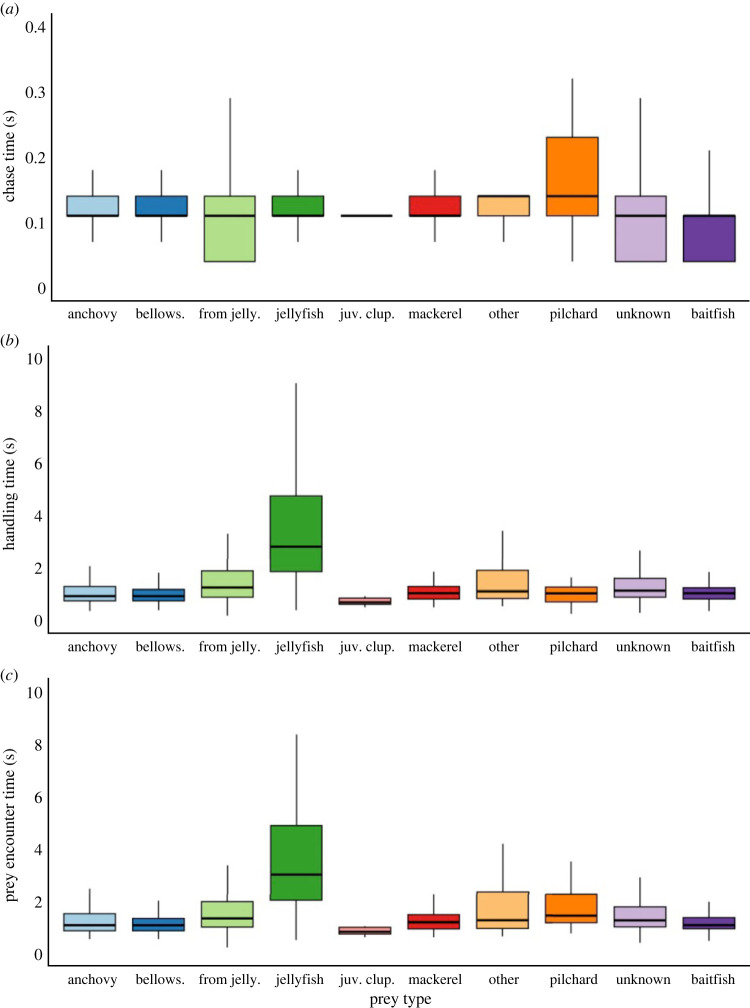
Boxplots summarizing (*a*) chase time, (*b*) handling time and (*c*) total prey encounter time spent by little penguins on different prey types in Bass Strait, Australia. Prey types are classified to the lowest taxonomic level identified. Further groupings include: from jelly—prey items extracted from jellyfish tentacles; juv. clup—juvenile fish from the order clupeiformes; other—prey types consumed less than 10 times; baitfish—fish that are unable to be identified by species level, but would fall into a small schooling fish order.

Prey encounter durations deviated from the reference line on the *Q*–*Q* plot. After transformation, the distribution improved but remained right-skewed, therefore, medians and maximums are reported (figure 2). Prey encounter durations ranged from 0.21 to 19.54 s. Median chase times ranged from 0.11 to 0.23 s among prey types, with a maximum of 10.54 s recorded. Median handling time ranged from 0.70 to 3.04 s among all prey types, up to a maximum of 19.39 s. Median prey encounter times ranged from 0.82 to 3.18 s, up to a maximum of 19.54 s. Median chase times were similar and short among all prey types. Pilchard had the longest median chase time (0.23 s) and was significantly different to all other prey types (Tukey's *post hoc* test, *p* < 0.001). Jellyfish had the longest median handling and total encounter time (3.04 s and 3.18 s, respectively), which was significantly longer than other prey types (Tukey's *post hoc* test, *p* < 0.001). Apart from jellyfish, all prey types had median handling and encounter durations of less than 2 s (table 2).

Two candidate models explaining prey encounter time included prey type only or prey type and BW (electronic supplementary material, table S1). Sex as well as the remaining morphometrics (body mass, FL, BL and HL) were not included in these candidate models, indicating no obvious influence on prey encounter time. Model averaging indicated the most parsimonious model to include prey type and BW (table 3). While model averaging retained BW as a predictor, confidence intervals for this parameter crossed zero revealing no consistent effect. There was no obvious effect of individual ID (*τ*_00_ = 0.01) on prey encounter time.

**Table 3.** Model average coefficient estimates (CEs) for the factors influencing prey encounter time in little penguins. Italicized parameter estimates indicate those for which the 95% confidence interval did not cross zero.

response variable	predictor effect	CE ± s.e.	*z*-value (*p*)	CI 2.75%	97.5%
prey encounter time	*intercept*	0.79 ± 0.20	3.90 (<0.001)	0.39	1.19
	prey type—bellowsfish	−0.04 ± 0.03	1.73 (0.08)	−0.09	0.01
	*prey type*—*CFJ*	0.15 ± 0.03	5.41 (<0.001)	0.10	0.21
	*prey type*—*jellyfish*	0.70 ± 0.03	22.31 (<0.001)	0.64	0.76
	*prey type*—*juvenile clupeiform*	−0.23 ± 0.08	2.76 (0.006)	−0.40	−0.07
	prey type—mackerel	0.04 ± 0.04	1.05 (0.29)	−0.04	0.12
	prey type—other prey	0.12 ± 0.07	1.62 (0.11)	−0.03	0.26
	*prey type*—*pilchard*	0.20 ± 0.10	2.09 (0.04)	0.01	0.39
	prey type—unidentifiable	0.04 ± 0.03	1.40 (0.16)	−0.01	0.09
	prey type—unidentifiable baitfish	−0.03 ± 0.03	1.03 (0.30)	−0.09	0.03
	bill width	0.01 ± 0.03	0.41 (0.69)	−0.04	0.06

Prey encounter times were significantly different between some prey types and, therefore, were investigated through *post hoc* tests (electronic supplementary material, table S2). Notably, the prey encounter duration for pilchard and prey captured from jellyfish was significantly longer than encounter durations for both bellowsfish and unidentified baitfish (Tukey's *post hoc* test *p* = 0.011 and *p* = 0.018, *p* < 0.001 and *p* = 0.014, respectively). Encounter durations for all other prey types were not significantly different from one another.

### 3.3. Prey energetic outcomes

**Figure 3.**
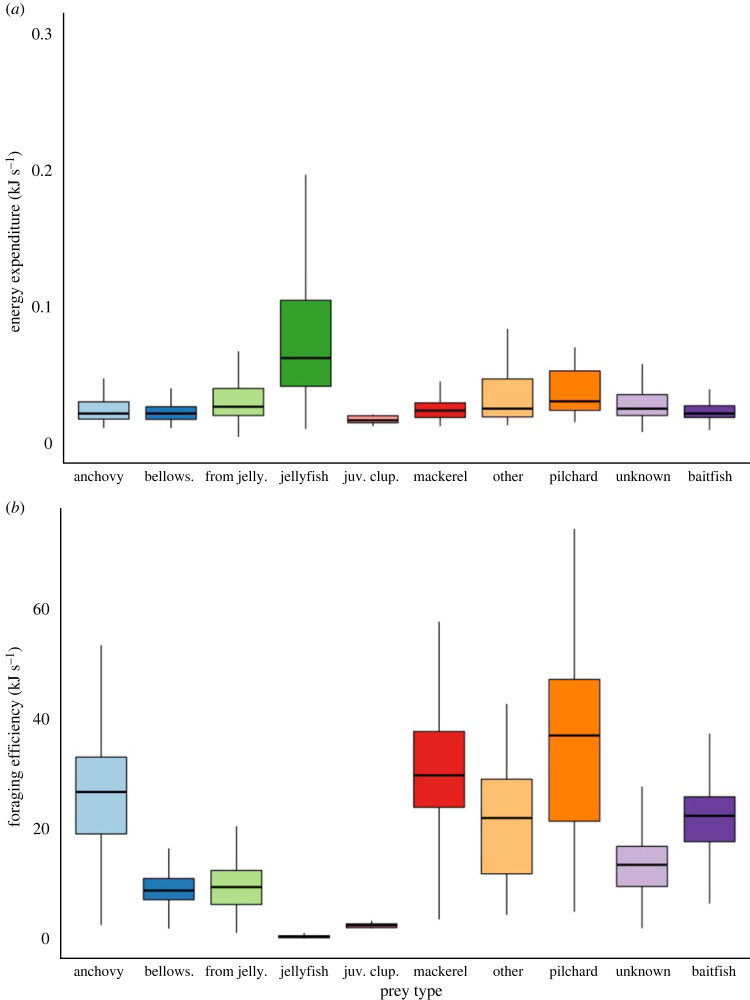
Boxplots summarizing (*a*) energy expenditure (kJ·s^−1^) and (*b*) Foraging efficiency (kJ·s^−1^) of little penguins for different prey types in Bass Strait, Australia. Prey types are classified to the lowest taxonomic level identified. Further groupings include: from jelly—prey items extracted from jellyfish tentacles; juv. clup—juvenile fish from the order clupeiformes; other—prey types consumed less than 10 times; baitfish—fish that are unable to be identified by species level, but would fall into a small schooling fish order.

The estimated nutritional value of prey types ranged between 1.2 kJ and 55.9 kJ (table 1). Jellyfish had the lowest nutritional value (1.2 kJ), along with the highest median energy expenditure (63.6 J s^−1^), making them the least profitable prey item (0.4 kJ s^−1^, figure 3). The most profitable prey for little penguins to capture were pilchard (36.9 kJ s^−1^), despite the energy expended being considerably high (30.4 J s^−1^). This was followed by anchovy and mackerel, both with intermediate profitability (26.7 kJ s^−1^ and 29.7 kJ s^−1^, respectively). Although bellowsfish and prey captured from jellyfish were the two most consumed prey types, their profitability was considerably lower (8.7 kJ s^−1^ and 9.4 kJ s^−1^, respectively). While juvenile clupeiform were of significantly lower profitability (2.4 kJ s^−1^), they accounted for only less than 1% of all captures.

## 4. Discussion

Knowledge of how animals effectively exploit food is fundamental to understanding the decisions made to cope in environments where food availability fluctuates spatially and temporally. To be efficient, individuals should adjust their time allocated to foraging behaviours (e.g. pursuing and handling prey) to maximize energy intake per unit time [55]. Here we used animal-borne cameras to investigate prey encounter durations and the associated profitability in little penguins. The results revealed prey type, but not intrinsic factors (sex and morphometrics), influenced encounter durations. Although highly profitable prey was consumed, little penguins consumed greater quantities of low profitable prey. This may indicate a trade-off between the energetic costs of chasing and handling prey and profitability of prey encounter.

### 4.1. Intrinsic and extrinsic factors influencing prey encounter duration

Prey characteristics are the main influence on prey handling duration in many other predators, with prey size being most influential for some species [56,57] while prey energy content is more influential in others [3,8]. In the present study, prey type influenced prey encounter duration with the prey types consumed varying in both size and energy content. This variation in prey type is expected as the water surrounding both the colonies in this study display varying degrees of productivity and are oceanographically different. LB is located near the seasonally active Bonney Upwelling, which makes the water highly productive. By contrast, GI waters are fed by eddies from the East Australian Current, which brings high nutrient concentrations and large biodiversity to these waters [28,58].

In the current study, some of the prey types frequently observed were schooling fish (anchovy, mackerel, pilchard), which is consistent with the known prey of little penguins in Bass Strait [13,49]. Yet surprisingly, bellowsfish were the most consumed prey in the present study even though they have not previously been recorded being consumed by little penguins. From the camera footage, it is assumed that the bellowsfish captured were pelagic juveniles which are known to swim in large schools near the surface [41]. Bellowsfish are a common larval fish found over the south-eastern Australian shelf along with many other larval species including clupeids and mackerels [59]. As bellowsfish occur in the same regions as other prey species commonly targeted by little penguins, it is likely that their consumption has been overlooked in previous diet studies [13,14,26,49,60].

As has recently been observed in other studies [34,60], jellyfish were consumed by little penguins in the present study. While it was not always possible to determine whether the penguins consumed the whole jellyfish, the inner portions or whether they were extracting prey from the tentacles, the frequency at which jellyfish were targeted suggests an importance in the diet of little penguins [61]. Interestingly, many prey encounters involved fish being captured near jellyfish. The majority of these were juvenile fish sheltering around jellyfish [62]. While prey captures from jellyfish have been identified in another seabird species [63], the use of a larger conspicuous prey source to indicate the presence of other smaller, more cryptic prey has not been previously identified in little penguins before. Other than being a source of prey, it is evident that jellyfish provide a refugia for small fish and may be an indicator of additional energetically richer prey. Thus, potentially reducing the effort involved in searching for and chasing smaller prey.

As the prey consumed are mobile, search times and dive depth may influence the overall profitability of certain prey types [64]. Despite their importance, due to limitations of the video data, these metrics were not possible to include in the present study. In addition, short chase durations may have been biased for some prey events as a change in direction (intentioned movements) and an increase in swimming speed were not always evident in the videos. Many prey events did not include a chase time as it was not always visually possible to distinguish these intentioned movements and regular swimming behaviour without observing flipper beat frequency or speed measurements. However, the generally short chase durations may have been due to individuals targeting bait balls. In such cases, a single search/chase duration would be associated with arriving at the bait ball and, for all subsequent captures at the bait ball, chases would be brief.

All prey, excluding jellyfish, had median encounter durations of less than 2 s suggesting little penguins spend little time chasing and handling prey. The most consumed prey types, accounting for over half of all observed captures, were mainly smaller schooling or juvenile fish. Optimal foraging theory suggests that predators should select prey types that can be collected most economically [65]. The swimming speed of a juvenile baitfish is approximately one tenth that of an adult fish [13] and, thus, it is possible that little penguins prefer targeting juvenile fish, finding them easier to capture. Indeed, a recent study demonstrated that little penguins spent longer periods at patches of juvenile prey [35]. While this prey type may be of lower energetic value, it may make for an efficient meal as it occurs in large schools and has shorter handling times in comparison to other prey.

Little penguins are sexually dimorphic [39] which may lead to differences in foraging behaviours, with swimming speeds, dive depths and durations more substantial in males [30,54]. Surprisingly, in the present study, there was no significant influence of sex or morphometrics on prey handling durations. The lack of influence from intrinsic variables could be related to the narrow spatial and temporal window in which individuals must forage before returning to the colony. If there were substantial prey within proximity to the colony and at shallow depths both males and females should exhibit similar foraging behaviours. Indeed, males and females during the early stages of chick-rearing have shown no difference in the composition of their diet [24,29,34].

### 4.2. Energetic profitability of prey

Little penguins may need to adjust their feeding strategies to cope with diet variation due to food resource availability and their breeding stage [38]. Feeding strategies could be the consequence of proactive decisions to increase energetic and nutritional requirements as a consequence of such variation [27]. Earlier studies assessing prey encounter time demonstrate that individuals select prey based upon maximizing net energy intake [3,66]. In the present study, individuals consumed a range of prey types with varying associated energy contents. This might reflect the lack of availability of high energy prey within the environment and the generalist nature of little penguins [12,14]. Prey encounter durations and, therefore, the energetic consequences were prey-specific. For example, the estimated energy gain from individual bellowsfish was relatively low. However, its short chase and handling duration, and schooling nature, would mean its capture rate could be comparatively high, allowing individuals to rapidly exploit prey patches. Indeed, the prominence of bellowsfish in the present study would suggest that little penguins may rely on these fish as an important prey item, given their high capture rate and abundance [35]. By contrast, while the estimated gross energy gain from jellyfish was also relatively low, their longer handling time (possibly due to their larger size) contributed to their low energetic profitability. However, whereas most of the adult bait fish observed had slightly longer encounter durations, they were still energetically profitable due to their larger size and high energy content. The results of the present study suggest that, in the absence of high energy prey, smaller sized schooling prey could be just as energetically profitable as they provide significant return for shorter encounter times.

Chick-rearing is a crucial phase for seabirds as adults need to return frequently to supply their offspring with adequate food [67]. Central place foragers face time and energy restrictions in searching for food and, therefore, must be proficient at locating and handling it. Little penguins are known to consume high quality prey items such as anchovy and mackerel to maximize rate of energy provisioning of offspring [29,38]. In the present study, penguins were tracked in the breeding season where they were foraging for self-maintenance and offspring provisioning. Of the prey captured, pilchard, anchovy and mackerel provided the highest rate of energy gain but represented a small proportion of recorded captures. This suggests that penguins may cope with the highly demanding chick-rearing phase by selecting profitable prey to feed their chicks when present, but seemed to typically select abundant prey with lower costs (i.e. search, pursuit and handling time) such as jellyfish and fish under jellyfish to ensure their chicks will be fed.

In summary, the present study revealed that prey type contributed to the variation in prey encounter time. The differences in duration contributed to the energetic costs and the profitability associated with prey consumption. Sex or morphology did not appear to influence encounter durations. However, due to the restrictions imposed upon adults in the chick-rearing stage, further investigation would be required to establish whether these factors could be influential outside of the breeding season. Highly profitable prey were captured when available, however, little penguins consumed a greater amount of low- to intermediate-profitable prey, generally associated with shorter encounter durations. This may enable individuals to consume more prey items with minimal energetic costs. Variation in prey types and encounter durations highlights how the generalist foraging behaviour of little penguins alters in response to prey availability which is ultimately influenced by oceanographic characteristics.

## Data Availability

Data available from the Dryad Digital Repository: https://doi.org/10.5061/dryad.5qfttdz9d [68]. Additional information is provided in the electronic supplementary material [69].
